# Morphological and phylogenetic analyses reveal new species of the *Fusarium lateritium* species complex and *Fusarium torreyae* species complex (*Nectriaceae*, *Hypocreales*) from Hainan Province, China

**DOI:** 10.3897/mycokeys.141.209463

**Published:** 2026-09-29

**Authors:** Jiawang Zhang, Yaling Wang, Jinyang Yuan, Xiuguo Zhang

**Affiliations:** 1 College of Plant Protection, Shandong Agricultural University, Taian, Shandong, 271018, China College of Plant Protection, Shandong Agricultural University Taian China

**Keywords:** *
Fusarium
*, morphology, multigene phylogeny, novel taxa

## Abstract

During a survey of fungi associated with woody plants in Hainan Province, China, two novel *Fusarium* species were discovered from symptomatic leaves of *Castanopsis* sp. and *Mallotus japonicus*. Through morphological examination and multi-locus phylogenetic analyses, the isolate from *Mallotus japonicus* was identified as a new species within the *Fusarium lateritium* species complex (FLSC) based on *tef1*, *tub2*, *rpb2*, and *CaM* sequences, and is described here as *Fusarium malloti***sp. nov**. In contrast, the isolate from *Castanopsis* sp. was resolved as a distinct lineage within the *Fusarium torreyae* species complex (FtorSC), supported by ITS, LSU, *rpb2*, *rpb1*, and *tef1* sequence data and is described as *Fusarium castanopsidicola***sp. nov**. Both new species are morphologically characterized and phylogenetically distinct from their closest relatives. These findings highlight the significant but underexplored diversity of *Fusarium* in Hainan and reinforce the necessity of integrating morphological characters with multi-locus phylogenetic analyses for accurate species delimitation in this genus.

## Introduction

*Fusarium* was established by Link (1809) to accommodate fungi characterized by the production of elongated, fusoid, multiseptate, falcate macroconidia. *Fusarium* is one of the most ecologically diverse and intensively studied fungal genera, with species occupying various niches as saprobes, endophytes, or pathogens. Many of these species pose serious threats to plants, humans, and animals by causing diseases or producing mycotoxins, thereby impacting global food security and safety (Leslie and Summerell 2006; Wu 2007; Dean et al. 2012; Proctor et al. 2013; Aoki et al. 2014; Costa et al. 2024; Xiao et al. 2025). Accurate identification of *Fusarium* species is essential for disease diagnosis and management. However, such identification is challenged by overlapping conidial morphology and inconsistent taxonomic frameworks based on either morphological or phylogenetic criteria (Han et al. 2023; Ai et al. 2025).

At present, *Fusarium* comprises 20 recognized species complexes and three undefined or unclear complexes (https://www.fusarium.org/). Among them, *Fusarium lateritium* holds a historically significant position within the genus. The name *Fusarium lateritium* was first proposed by Nees in 1817, based on a collection of *Fagus* twigs. In 1917, Wollenweber established Section *Lateritium* for species with pale- to rose-colored cultures, blue erumpent sclerotia, intercalary (not terminal) chlamydospores, and relatively straight macroconidia with a curved, often beaked apical cell and a pedicellate foot-cell. O’Donnell et al. (2013) delimited the *Fusarium lateritium* species complex (FLSC) based on a multi-locus phylogeny, initially placing three species within it: *Fusarium lateritium*, *Fusarium stilboides*, and *Fusarium sarcochroum*. The complex has since grown substantially, with 29 phylogenetic species currently accepted, many of which have been recovered from woody plants (Geiser et al. 2005; Cavalcanti et al. 2020; Perera 2020; Crous et al. 2022; Hyde et al. 2023; Costa et al. 2024; He et al. 2024). The *Fusarium torreyae* species complex (FtorSC) was established based on comparative studies of canker pathogens from *Torreya taxifolia* in Florida and *Zanthoxylum bungeanum* in China and comprises three core species: *F. torreyae*, *F. zanthoxyli*, and *F. continuum* (Zhou et al. 2016). Subsequently, Tan et al. (2025) discovered *Fusarium verae* from soil in New South Wales, Australia (Maryland River), and the FtorSC now comprises four species.

In this study, two new species were discovered from Hainan Province, China. Based on morphological examination and multi-locus phylogenetic analyses, they were identified as new members of the FLSC and FtorSC, respectively.

## Materials and methods

### Strain isolation and preservation

In Hainan Province, plant specimens with diseased leaves were collected during 2023–2024. Collected samples were labeled with the date, locality, and host species and subsequently photographed and documented. Tissue isolation techniques were used to obtain pure colonies. Approximately five to seven infected or decaying leaf fragments (5 × 5 mm) were excised from the lesion margins of each sample and surface-sterilized by sequential immersion in 75% ethanol (1 min), sterile water (30 s), and 5% sodium hypochlorite solution (1 min). The fragments were rinsed three times with sterile water (30 s each), dried on sterile filter paper, and then placed on potato dextrose agar (PDA) plates and incubated at 25 °C in the dark for 3 days. Hyphae from the colony margins were transferred to fresh PDA plates. The resulting pure isolates were maintained in 15% sterile glycerol and stored at 4 °C for further analysis. Voucher specimens are preserved in the Herbarium of the Department of Plant Pathology, Shandong Agricultural University, Tai’an, China (HSAUP). Corresponding ex-type living cultures are preserved in two culture collections: the Shandong Agricultural University Culture Collection (SAUCC) and the China General Microbiological Culture Collection Center (CGMCC).

### Morphological characterization

After 7 days of incubation on PDA, colony morphology, pigmentation, and growth rates were recorded, and the plate surfaces (obverse and reverse) were documented using a Canon Powershot G7X digital camera (Canon, Tokyo, Japan). Morphological features, including conidial shape, size, and septation, were described from cultures grown on water agar (WA) with carnation leaves. Microstructures were examined using an Olympus SZ61 stereomicroscope and an Olympus BX53 compound microscope equipped with differential interference contrast (DIC). Fungal microstructures were photographed using BioHD-A20c color digital cameras (FluoCa Scientific, Shanghai, China) attached to both the stereomicroscope and the compound microscope. Microstructures were randomly measured using Digimizer software v. 5.6.0, and their average size (av.) was computed. The number of measurements is denoted by “*n*”.

### DNA extraction, amplification, and sequencing

The cetyltrimethylammonium bromide (CTAB) method was employed to extract total genomic DNA from fungal mycelia grown on PDA for 7 days (Damm et al. 2008). Primer details and their corresponding references are listed in Table 1. The 20 µL polymerase chain reaction (PCR) mixture comprised 10 µL of 2 × Hieff Canace® Plus Master Mix, 0.5 µL each of the forward and reverse primers (10 µM; TsingKe, Qingdao, China), 1 µL of genomic DNA template, and nuclease-free water to volume. Amplified products were electrophoresed on a 1% agarose gel stained with GelStain and visualized under UV light. Target DNA bands were excised from the gel and purified with a gel extraction kit (DM1200, Beijing Solarbio Science & Technology Co., Ltd.). Purified PCR products were sequenced by TsingKe Company Limited (Qingdao, China). Sequence data were analyzed using MEGA v. 7.0 (Kumar et al. 2016), and all newly generated sequences were deposited in GenBank (https://www.ncbi.nlm.nih.gov/) (Tables 2, 3).

**Table 1.** Molecular markers and their corresponding PCR primers and programs used in this study.

**Loci**	**PCR primers**	**Sequence (5'→3')**	**PCR cycles**	**References**
*tef1*	EF1	ATG GGT AAG GAR GAC AAG AC	95 °C 5 min; (94 °C 45 s, 55 °C 45 s, 72 °C 15 min) × 35 cycles	O’Donnell et al. 1998, 2008
	EF2	GGA RGT ACC AGT SAT CAT GTT		
*rpb2*	5f2	GGG GWG AYC AGA AGA AGG C	95 °C 5 min; (94 °C: 45 s, 55 °C: 45 s, 72 °C: 15 s) × 35 cycles	Liu et al. 1999; Reeb et al. 2004
	7cf	ATGGGYAARCAAGCYATGGG		
	7cr	CCC ATR GCT TGY TTR CCC AT		
	11ar	GCRTGGATCTTRTCRTCSACC		
*tub2*	Bt - 2a	GGT AAC CAA ATC GGT GCT GCT TTC	95 °C 5 min; (95 °C: 30 s, 53 °C: 30 s, 72 °C: 1 min) × 35 cycles	Glass and Donaldson 1995
	Bt - 2b	ACC CTC AGT GTA GTG ACC CTT GGC		
*CaM*	CL1	GARTWCAAGGAGGCCTTCTC	(94 °C: 30 s, 55 °C: 30 s, 72 °C: 15 s) × 35 cycles	O’Donnell et al. 2020
	CL2A	TTTTTGCATCATGAGTTGGAC		
ITS	ITS5	GGAAGTAAAAGTCGTAACAAGG	(95 °C 30 s, 52 °C 30 s, 72 °C 10 s) × 35 cycles	White et al. 1990
	ITS4	TCCTCCGCTTATTGATATGC		
*rpb1*	F7	CRACACAGAAGAGTTTGAAGG	(95 °C 2 min, 58 °C 45 s, 72 °C 20 s) × 5 cycles; (95 °C 2 min, 57 °C 45 s, 72 °C 20 s) × 5 cycles; (95 °C 2 min, 56 °C 45 s, 72 °C 20 s) × 35 cycles	O’Donnell et al. 2010
	G2R	GTCATYTGDGTDGCDGGYTCDCC		
LSU	LR0R	GTA CCC GCT GAA CTT AAG C	(95 °C 30 s, 52 °C 30 s, 72 °C 1 min) × 35 cycles	Vilgalys and Hester 1990; Rehner and Samuels 1994
	LR5	TCC TGA GGG AAA CTT CG		

**Table 2.** GenBank accession numbers of taxa used in the phylogenetic reconstruction of the FLSC.

**Species name**	**Strain code**	**GenBank accession numbers**				
		***tef1***	***tub2***	***rpb2*-part 1**	***rpb2*-part 2**	***CaM***
*Fusarium aurantii*	BBA 65609 = CBS 152039 = CPC 44982 = DSM 115149^*^	PQ000452	PQ000532	PQ000617	PQ000695	PQ000774
*F. aurantii*	FRC L-200	AY707168	AY707150	NA	NA	NA
*F. aurantii*	IMI 310541 = CPC 44539	PQ000453	PQ000533	PQ000618	PQ000696	PQ000775
*F. aurantii*	IMI 338937 = CPC 42982	PQ000454	PQ000534	PQ000619	PQ000697	PQ000776
*F. camelliae*	GUCC 21010^*^	OP757314	NA	OP800100	NA	NA
*F. camelliae*	GUCC 21011	OP800102	NA	OP820349	NA	NA
*F. cartwrightiae*	BRIP 74113d = CBS 150776^*^	OR335749	PQ000582	OR335732	NA	PQ000824
*F. cassiae*	BBA 65092 = CPC 47105 = DSM 115143	PQ000502	PQ000583	PQ000666	PQ000744	PQ000825
*F. cassiae*	BBA 72582 = CPC 47273 = DSM 116938	PQ000503	PQ000584	PQ000667	PQ000745	PQ000826
*F. cassiae*	BBA 72583 = CPC 47274 = DSM 116939	PQ000504	PQ000585	PQ000668	PQ000746	PQ000827
*F. cassiae*	BBA 72584 = CPC 47275 = DSM 116940	PQ000505	PQ000586	PQ000669	PQ000747	PQ000828
*F. cassiae*	LC13727 = F092	MW594307	MW533911	MW474540	MW474540	NA
*F. cassiae*	MFLUCC 18-0573^*^	MT212205	NA	MT212197	NA	NA
*F. chlamydocopiosum*	CBS 152063 = CPC 37116^*^	PQ000506	PQ000587	PQ000670	PQ000748	PQ000829
*F. chlamydocopiosum*	CBS 152064 = CPC 37118	PQ000507	PQ000588	PQ000671	PQ000749	PQ000830
*F. chlamydocopiosum*	CBS 152065 = CPC 37119	PQ000508	PQ000589	PQ000672	PQ000750	PQ000831
*F. citri-sinensis*	BBA 67503 = CBS 152066 = CPC 44985 = DSM 115153^*^	PQ000509	PQ000590	PQ000673	PQ000751	PQ000832
*F. citri-sinensis*	YZU 191316	MW855826	NA	MW855854	NA	NA
*F. citri-sinensis*	YZU 181391	MW855825	NA	NA	NA	NA
*F. coffeibaccae*	BBA 62428 = CPC 46655 = DSM 115998	PQ000455	PQ000535	PQ000620	PQ000698	PQ000777
*F. coffeibaccae*	BBA 62429 = CPC 46656 = DSM 115999	PQ000456	PQ000536	PQ000621	PQ000699	PQ000778
*F. coffeibaccae*	BBA 62430 = CPC 46657 = DSM 116000	PQ000457	PQ000537	PQ000622	PQ000700	PQ000779
*F. coffeibaccae*	CBS 178.31 = NRRL 20838	PQ000458	PQ000538	PQ000623	PQ000701	PQ000780
*F. coffeibaccae*	CBS 101890	PQ000459	PQ000539	PQ000624	PQ000702	PQ000781
*F. coffeibaccae*	CBS 101891	PQ000460	PQ000540	PQ000625	PQ000703	PQ000782
*F. coffeibaccae*	CBS 101892	PQ000461	PQ000541	PQ000626	PQ000704	PQ000783
*F. coffeibaccae*	CBS 119871 = BBA 62458 = DSM 62458 = MRC 1845	PQ000462	PQ000542	PQ000627	PQ000705	PQ000784
*F. coffeibaccae*	CBS 119872 = MRC 2465	PQ000463	PQ000543	PQ000628	PQ000706	PQ000785
*F. coffeibaccae*	CML 90 = CBS 152040 = CPC 46658^*^	PQ000464	PQ000544	PQ000629	PQ000707	PQ000786
*F. coffeibaccae*	CML 178 = CPC 46661	PQ000465	PQ000545	PQ000630	PQ000708	PQ000787
*F. coffeibaccae*	CML 2400 = CBS 152041 = CPC 46659	PQ000466	PQ000546	PQ000631	PQ000709	PQ000788
*F. coffeibaccae*	CML 4403 = CBS 152042 = CPC 46662	PQ000467	PQ000547	PQ000632	PQ000710	PQ000789
*F. coffeibaccae*	CML 4404 = CBS 152043 = CPC 46663	PQ000468	PQ000548	PQ000633	PQ000711	PQ000790
*F. coffeibaccae*	CML 4405 = CBS 152044 = CPC 46664	PQ000469	PQ000549	PQ000634	PQ000712	PQ000791
*F. coffeibaccae*	CML 4407 = CBS 152045 = CPC 46666	PQ000470	PQ000550	PQ000635	PQ000713	PQ000792
*F. coffeibaccae*	CML 4408 = CBS 152269 = CPC 46667	PQ000471	PQ000551	PQ000636	PQ000714	PQ000793
*F. coffeibaccae*	CML 4409 = CBS 152046 = CPC 46668	PQ000472	PQ000552	PQ000637	PQ000715	PQ000794
*F. coffeibaccae*	CML 4410 = CBS 152047 = CPC 46669	PQ000473	PQ000553	PQ000638	PQ000716	PQ000795
*F. coffeibaccae*	CML 4411 = CBS 152048 = CPC 46670	PQ000474	PQ000554	PQ000639	PQ000717	PQ000796
*F. coffeibaccae*	CML 4414 = CBS 152049 = CPC 46671	PQ000475	PQ000555	PQ000640	PQ000718	PQ000797
*F. coffeibaccae*	CML 4415 = CBS 152050 = CPC 46672	PQ000476	PQ000556	PQ000641	PQ000719	PQ000798
*F. coffeibaccae*	CML 4423 = CBS 152051 = CPC 46677	PQ000477	PQ000557	PQ000642	PQ000720	PQ000799
*F. coffeibaccae*	CML 4424 = CBS 152052 = CPC 46678	PQ000478	PQ000558	PQ000643	PQ000721	PQ000800
*F. coffeibaccae*	CML 4427 = CBS 152053 = CPC 46679	PQ000479	PQ000559	PQ000644	PQ000722	PQ000801
*F. coffeibaccae*	CML 4428 = CBS 152054 = CPC 46680	PQ000480	PQ000560	PQ000645	PQ000723	PQ000802
*F. coffeibaccae*	CML 4430 = CBS 152055 = CPC 46681	PQ000481	PQ000561	PQ000646	PQ000724	PQ000803
*F. coffeibaccae*	CML 4431 = CBS 152056 = CPC 46682	PQ000482	PQ000562	PQ000647	PQ000725	PQ000804
*F. coffeibaccae*	CML 4432 = CBS 152057 = CPC 46683	PQ000483	PQ000563	PQ000648	PQ000726	PQ000805
*F. coffeibaccae*	CML 4435 = CBS 152058 = CPC 46685	PQ000484	PQ000564	PQ000649	PQ000727	PQ000806
*F. coffeibaccae*	CML 4436 = CBS 152059 = CPC 46686	PQ000485	PQ000565	PQ000650	PQ000728	PQ000807
*F. coffeibaccae*	CML 4437 = CBS 152274 = CPC 46687	PQ000486	PQ000566	PQ000651	PQ000729	PQ000808
*F. coffeibaccae*	CML 4438 = CBS 152060 = CPC 46688	PQ000487	PQ000567	PQ000652	PQ000730	PQ000809
*F. coffeibaccae*	CML 4440 = CBS 152061 = CPC 46690	PQ000488	PQ000568	PQ000653	PQ000731	PQ000810
*F. coffeibaccae*	CML 4443 = CBS 152062 = CPC 46691	PQ000489	PQ000569	PQ000654	PQ000732	PQ000811
*F. coffeibaccae*	FRC L-69	AY707155	AY707137	NA	NA	NA
*F. coffeibaccae*	FRC L-83	AY707158	AY707140	NA	NA	NA
*F. coffeibaccae*	FRC L-84	AY707159	AY707141	NA	NA	NA
*F. coffeibaccae*	FRC L-86	AY707160	AY707142	NA	NA	NA
*F. coffeibaccae*	FRC L-87	AY707161	AY707143	NA	NA	NA
*F. coffeibaccae*	FRC L-101 = BBA 62455	AY707163	AY707145	NA	NA	NA
*F. coffeibaccae*	FRC L-107 = MRC 1926	AY707164	AY707146	NA	NA	NA
*F. coffeibaccae*	FRC L-375	AY707169	AY707151	NA	NA	NA
*F. coffeibaccae*	FRC L-376	AY707170	AY707152	NA	NA	NA
*F. coffeibaccae*	FRC L-402	AY707171	AY707153	NA	NA	NA
*F. coffeibaccae*	IMI 194631 = CPC 42976	PQ000490	PQ000570	PQ000655	PQ000733	PQ000812
*F. coffeibaccae*	IMI 275737 = CPC 44569	PQ000491	PQ000571	PQ000656	PQ000734	PQ000813
*F. coffeibaccae*	IMI 312020 = CPC 42980	PQ000492	PQ000572	PQ000657	PQ000735	PQ000814
*F. coffeibaccae*	IMI 328353 = CPC 42981	PQ000493	PQ000573	PQ000658	PQ000736	PQ000815
*F. coffeibaccae*	IMI 343160 = CPC 42983	PQ000494	PQ000574	PQ000659	PQ000737	PQ000816
*F. coffeibaccae*	IMI 343468 = CPC 42984	PQ000495	PQ000575	PQ000660	PQ000738	PQ000817
*F. coffeibaccae*	IMI 375911 = CPC 42985	PQ000496	PQ000576	PQ000661	PQ000739	PQ000818
*F. coffeibaccae*	IMI 389578 = CPC 46697	PQ000497	PQ000577	PQ000662	PQ000740	PQ000819
*F. coffeibaccae*	IMI 389580 = CPC 46699	PQ000498	PQ000578	PQ000663	PQ000741	PQ000820
*F. coffeibaccae*	IMI 392282 = CPC 42972	PQ000499	PQ000579	PQ000664	PQ000742	PQ000821
*F. coffeibaccae*	IMI 393591 = CPC 44771	PQ000500	PQ000580	PQ000665	PQ000743	PQ000822
*F. coffeibaccae*	LC13732 = F085	MW594312	MW533916	MW474545	NA	NA
*F. coffeibaccae*	LC13733 = F088	MW594313	MW533917	MW474546	NA	NA
*F. coffeibaccae*	NRRL 20429 = ATCC 15662 = CBS 151960 = GORDON 4843	PQ000501	PQ000581	JX171582	JX171582	PQ000823
*F. crocatum*	BBA 65674 = CPC 44983 = DSM 115150	PQ000510	NA	PQ000674	PQ000752	PQ000833
*F. crocatum*	BBA 65926 = CBS 152067 = CPC 44984 = DSM 115152^*^	PQ000511	PQ000591	PQ000675	PQ000753	PQ000834
*F. crocatum*	CBS 130184 = BBA 65687 = DSM 115151 = NRRL 25197	PQ000512	PQ000592	PQ000676	PQ000754	PQ000835
*F. endophyticum*	CBS 319.73 = ATCC 24391 = CPC 42975 = FRC L-403 = IMI 155795 = NRRL 25482	PQ000513	PQ000593	PQ000677	PQ000755	PQ000836
*F. endophyticum*	CML 4473 = CBS 152068 = CPC 46692	PQ000514	PQ000594	PQ000678	PQ000756	PQ000837
*F. endophyticum*	FRC L-82	AY707157	AY707139	NA	NA	NA
*F. endophyticum*	FRC L-120	AY707167	AY707149	NA	NA	NA
*F. endophyticum*	IMI 373356 = CPC 46708	PQ000515	PQ000595	PQ000679	PQ000757	PQ000838
*F. endophyticum*	SDBR-CMU465^*^	OQ686616	NA	OQ686618	NA	NA
*F. endophyticum*	SDBR-CMU466	OQ686617	NA	OQ686619	NA	NA
*F. fujianense*	CFCC 57576 = LC14^*^	ON734389	ON734409	ON734369	NA	NA
*F. fujianense*	LC14-1	ON734390	ON734410	ON734370	NA	NA
*F. fujianense*	LC14-2	ON734391	ON734411	ON734371	NA	NA
*F. geijerae*	BRIP 64537a^*^	PV091689	NA	NA	NA	NA
*F. gummosum*	ZJUE1694	NA	NA	PX437981	PX437981	NA
*F. gummosum*	ZJUE1738^*^	NA	NA	PX437982	PX437982	NA
*F. gummosum*	ZJUE1739	NA	NA	PX437983	PX437983	NA
*F. hanswilhelmii*	CBS 633.76 = BBA 63665 = DSM 115136 = NRRL 25484^*^	PQ000516	PQ000596	PQ000680	PQ000758	PQ000839
*F. lateritium*	CBS 127477 = HMAS 86477	PQ000517	PQ000597	PQ000681	PQ000759	PQ000840
*F. lateritium*	NRRL 13622 = ATCC 60188 = CBS 151946 = FRC L-55 = NRRL A-26433^*^	PQ000518	PQ000598	JX171571	JX171571	PQ000841
*F. magnoliae-champaca*	MFLUCC 18-0580^*^	NA	NA	MT212198	NA	NA
*F. malawiense*	CBS 737.74 = ATCC 24375 = CPC 42954 = IMI 158154 = NRRL 20533^*^	PQ000519	PQ000599	PQ000682	PQ000760	PQ000842
***F. malloti***	SAUCC 3317C-1C = CGMCC3.40250 ^*^	**PZ695334**	**PZ748774**	**PZ777512**	**PZ777512**	**NA**
*F. massalimae*	FCCUFG 05	MN939764	MN939760	MN939768	NA	NA
*F. massalimae*	URM 8239^*^	MN939763	MN939759	MN939767	NA	NA
*F. microcyclum*	IMI 128389 = ATCC 24396 = CBS 137.73 = CPC 46696^*^	PQ000520	PQ000600	PQ000683	PQ000761	PQ000843
*F. microcyclum*	IMI 392677 = ATCC 36325 = CPC 44278 = DSM 62456 = FUS005	PQ000521	PQ000601	PQ000684	PQ000762	PQ000844
*F. oliniae*	CBS 152069 = CPC 38826^*^	PQ000522	PQ000602	PQ000685	PQ000763	PQ000845
*F. ramsdenii*	BRIP 62306a = CBS 150771^*^	OL332732	OL332734	OL332733	PQ000764	PQ000846
*F. rufum*	IMI 294142 = CPC 42977	PQ000523	PQ000603	PQ000686	PQ000765	PQ000847
*F. rufum*	IMI 300505 = CPC 43032^*^	PQ000524	PQ000604	PQ000687	PQ000766	PQ000848
*F. sarcochroum*	CBS 745.79 = BBA 63714 = NRRL 20472^*^	MW834278	PQ000605	JX171586	JX171586	PQ000849
*F. sarcochroum*	CBS 152070 = CPC 26369	LT746207	PQ000606	LT746320	LT746320	PQ000850
*F. sarcochroum*	CBS 152071 = CPC 26370	LT746208	PQ000607	LT746321	LT746321	PQ000851
*F. sarcochroum*	CBS 152072 = CPC 26851	LT746209	PQ000608	LT746322	LT746322	PQ000852
*F. sarcochroum*	CBS 152073 = CPC 27921	LT746210	PQ000609	LT746323	LT746323	PQ000853
*F. sarcochroum*	CBS 152075 = CPC 28116	LT746212	PQ000610	LT746325	LT746325	PQ000854
*F. stilboides*	BBA 65608 = CPC 44981 = DSM 115148	PQ000525	PQ000611	PQ000688	PQ000767	PQ000855
*F. stilboides*	CBS 746.79 = BBA 63887 = NRRL 25485^*^	MW928843	PQ000612	MW928832	MW928832	PQ000856
*F. stilboides*	CBS 115043 = HKUCC 6063	PQ000526	PQ000613	PQ000689	PQ000768	PQ000857
*F. stilboides*	FRC L-81	AY707156	AY707138	NA	NA	NA
*F. stilboides*	LC13728 = CQ1109	MW594308	MW533912	MW474541	NA	NA
*F. stilboides*	LC13729 = CQ993	MW594309	MW533913	MW474542	NA	NA
*F. stilboides*	LC13730 = F004	MW594310	MW533914	MW474543	NA	NA
*F. stilboides*	LC13731 = M0759	MW594311	MW533915	MW474544	NA	NA
*F. stramineum*	BBA 65675 = CBS 152076 = CPC 47569 = DSM 116943^*^	PQ000527	PQ000614	PQ000690	PQ000769	PQ000858
*Fusarium* sp.	FRC L-110	AY707165	AY707147	NA	NA	NA
*Fusarium* sp.	FRC L-112	AY707166	AY707148	NA	NA	NA
*F. velutinum*	CBS 256.93 = INIFAT C91/223-6F = NRRL 25490	PQ000528	PQ000615	PQ000691	PQ000770	PQ000859
*F. velutinum*	IMI 353670 = CPC 44738^*^	PQ000529	PQ000616	PQ000692	PQ000771	PQ000860
*F. verruculosum*	BBA 68498 = CPC 46464 = DSM 116556	PQ000530	NA	PQ000693	PQ000772	PQ000861
*F. verruculosum*	BBA 68499 = CBS 152077 = CPC 44987 = DSM 115156^*^	PQ000531	NA	PQ000694	PQ000773	PQ000862
*F. wenshanense*	KUNCC 3511	PV413188	PV476709	PV521353	PV521353	PV442466
*F. wenshanense*	KUNCC 3512 = HKAS 135098^*^	PV413189	PV476708	PV521352	PV521352	PV442465
*F. wenshanense*	KUNCC 3510	PV413190	PV476703	PV521351	PV521351	PV442464
*F. sublunatum*	CBS 189.34 = BBA 62431 = DSM 62431 = IMB 5238 = NRRL 13384 = NRRL 20840^*^	OM160871	KM232076	JX171565	JX171565	KM231389

Notes: Species established in this study are shown in bold. Those marked with an asterisk “*” in the table are represented by ex-type or ex-epitype strains. NA: Not available.

**Table 3.** GenBank accession numbers of taxa used in the phylogenetic reconstruction of the FtorSC.

**Species name**	**Strain code**	**ITS**	**LSU**	***rpb1***	***rpb2***	***tef1***
***F. castanopsidicola***	SAUCC14250-1 = CGMCC3.40253 ^*^	**PZ955447**	**PZ955448**	**PZ954595**	**PZ621803 PZ628922**	**PZ706463**
*F. continuum*	F201030 = NRRL 66286 = CBS 140841*	KM236752	MH878195	KM520387	KM236782	KM236722
*F. continuum*	F201128	KM236750	NA	KM520389	KM236780	KM236720
*F. continuum*	F201127	KM236749	NA	KM520386	KM236779	KM236719
*F. continuum*	F201126	KM236748	NA	KM520388	KM236778	KM236718
*F. continuum*	F201129	KM236751	NA	KM520385	KM236781	KM236721
*F. torreyae*	CBS 133858 = MAFF 243468 = NRRL 54151*	HM068346	MW827652	MW928819	MW928834	HM068339
*F. torreyae*	CBS 133201	HM068344	NA	NA	NA	HM068337
*F. verae*	MST F23626*	NA	NA	NA	PX400652	PX400653
*F. zanthoxyli*	F201311 = NRRL 66285 = CBS 140838*	OM117610	MH878194	KM520383	KM236763	KM236703
*F. zanthoxyli*	F201114	KM236736	NA	KM520380	KM236766	KM236706
*F. zanthoxyli*	F201313	KM236735	NA	NA	KM236765	KM236705
*F. zanthoxyli*	F201125	KM23674	NA	NA	KM236777	KM236717
*F. venenatum*	CBS 458.93	KM231814	MH874080	MW233256	MW233428	MW233085

Notes: Species established in this study are shown in bold. Those marked with an asterisk “*” in the table are represented by ex-type or ex-epitype strains. NA: Not available.

### Phylogenetic analyses

Sequence alignment was performed using the MAFFT v. 7 online service, and the resulting alignments were manually corrected in MEGA v. 7.0 (Katoh et al. 2019). The concatenated sequences were used for phylogenetic analyses. Maximum likelihood (ML) analysis was conducted in RAxML-HPC2 v. 8.2.12 with 1,000 bootstrap replicates and default parameters. Bayesian inference (BI) analysis was performed using MrBayes v. 3.2.7a on the Linux platform with Markov chain Monte Carlo (MCMC) algorithms, and the best-fit evolutionary model for each partition was determined using MrModeltest v. 2.3 (Nylander 2004). The phylogenetic trees were visualized and optimized using the Interactive Tree of Life (iTOL) platform (https://itol.embl.de/), with final layout adjustments made in Adobe Illustrator CS6 (Adobe Systems Inc., USA). Isolates obtained in this study are highlighted in red in the phylogenetic trees. Separate phylogenetic trees for the FLSC and FtorSC are also presented (Suppl. materials 1, 2).

## Results

### Phylogenetic analyses

**Figure 1.**
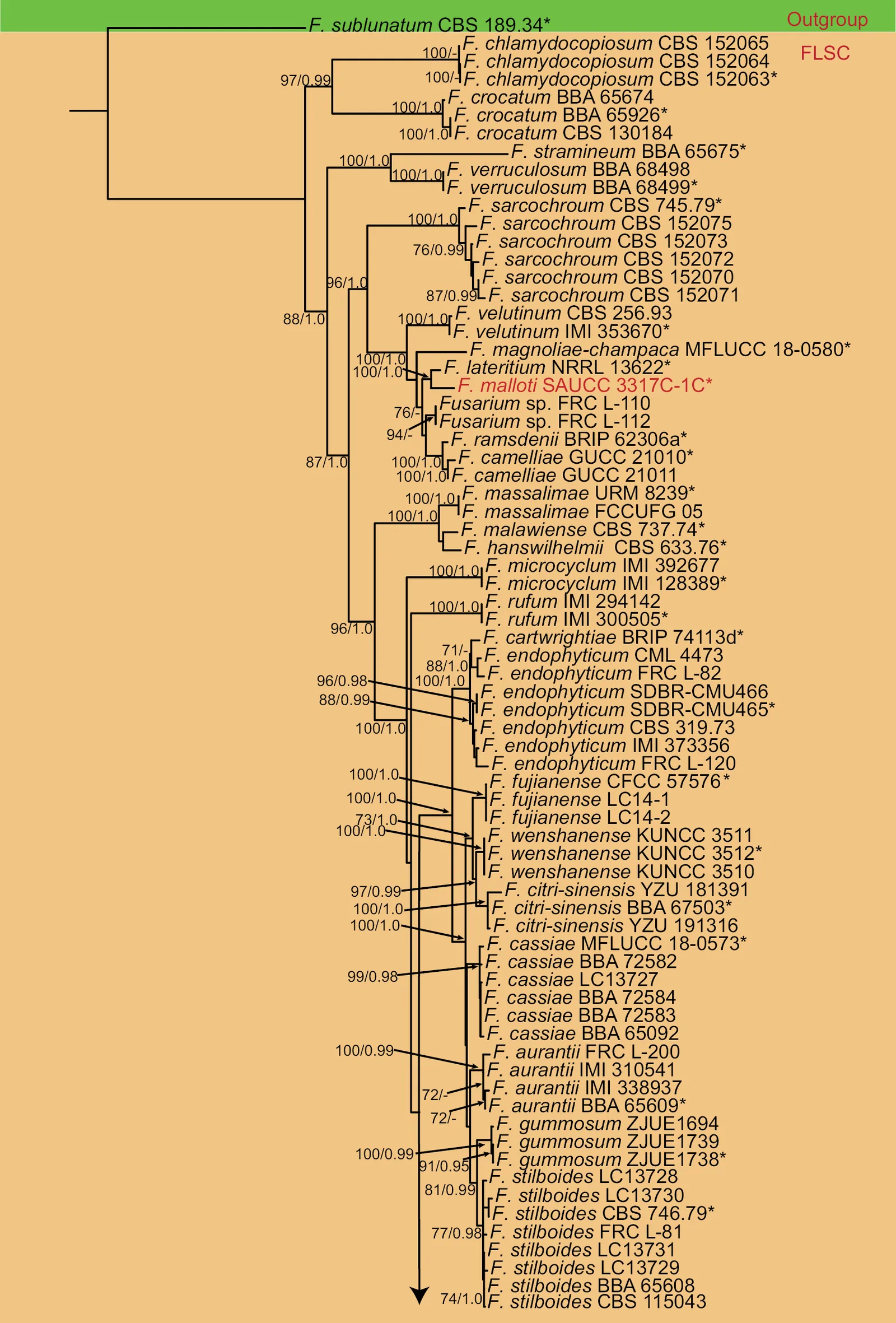
Phylogenetic tree inferred from a maximum likelihood (RAxML) analysis based on concatenated *tef1*, *tub2*, *rpb2*, and *CaM* sequences of species from the FLSC. Those marked with an asterisk (*) in the tree are represented by ex-type or ex-epitype strains. RAxML bootstrap support values (MLBS ≥ 70%) and Bayesian inference posterior probabilities (BIPP ≥ 0.90) are shown at the nodes. The tree was rooted with *Fusarium sublunatum* (CBS 189.34) as the outgroup. Strains isolated in this study are indicated in red. The figure shows partial branches of the evolutionary tree, and the full evolutionary tree is provided in Suppl. material 3.

In the phylogenetic tree of the FLSC containing 134 *Fusarium* isolates, the strain *Fusarium sublunatum* (CBS 189.34) served as the outgroup. The alignment comprised 3,102 concatenated nucleotide bases, viz., 1–510 (*tef1*), 511–853 (*tub2*), 854–2535 (*rpb2*), and 2536–3102 (*CaM*). Among these sequences, 2,191 characters were constant, 705 characters were variable and parsimony-uninformative, and 206 characters were parsimony-informative. The topologies obtained from the ML and BI analyses were consistent; therefore, the ML tree is shown in this study. The 134 tested strains were identified as 32 species based on the phylogenetic analysis of the *tef1*, *tub2*, *rpb2*, and *CaM* genes. In the BI analysis, the alignment contained 1,156 unique site patterns (*tef1*: 271; *tub2*: 337; *rpb2*: 321; *CaM*: 227). BI analysis was conducted for 4,435,000 generations, yielding 8,872 consensus trees (Fig. 1; BIPP ≥ 0.90 is displayed as the first value). In the phylogenetic tree, *Fusarium malloti* (SAUCC 3317C-1C) formed a well-supported clade (MLBS/BIPP = 100%/1.0) sister to *F. lateritium* (NRRL 13622) within the FLSC.

**Figure 2.**
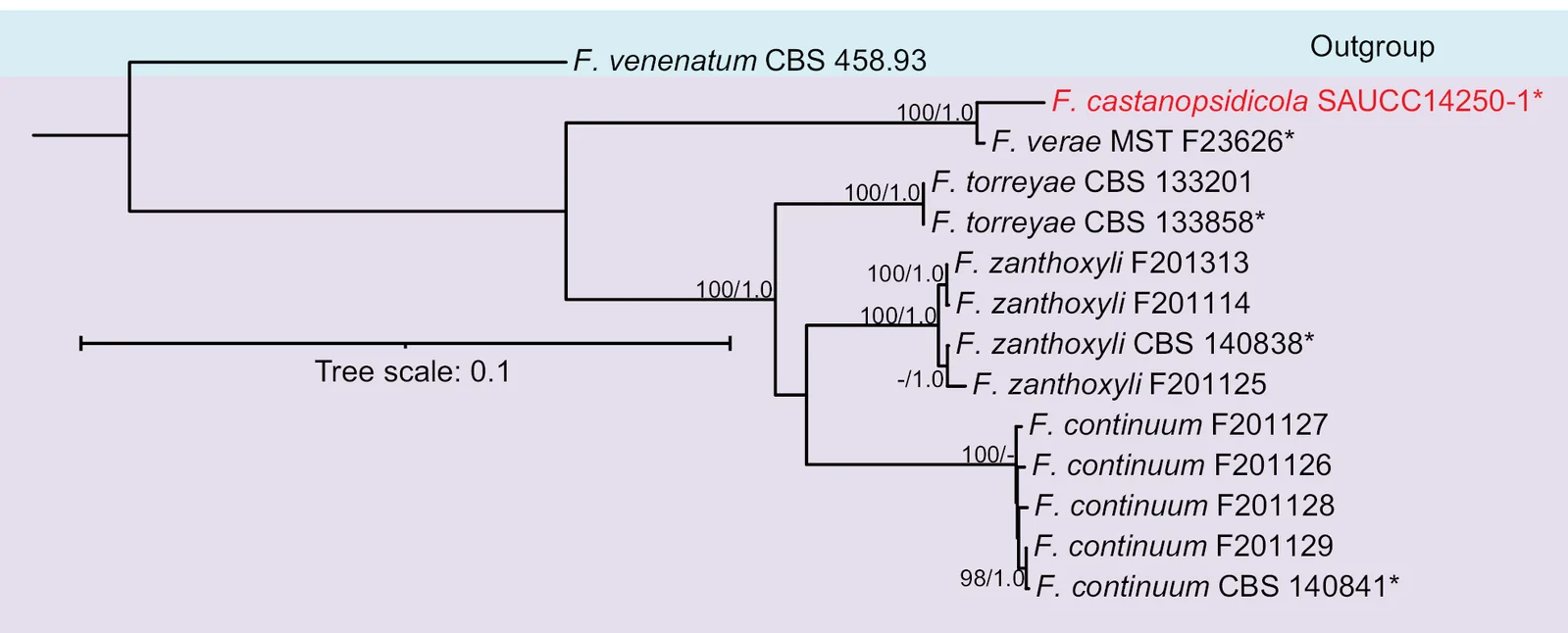
Phylogenetic tree inferred from a maximum likelihood (RAxML) analysis based on concatenated ITS, LSU, *rpb1*, *rpb2*, and *tef1* sequences of species from the FtorSC. Those marked with an asterisk (*) in the tree are represented by ex-type or ex-epitype strains. RAxML bootstrap support values (MLBS ≥ 80%) and Bayesian inference posterior probabilities (BIPP ≥ 0.95) are shown at the nodes. The tree was rooted with *Fusarium venenatum* (CBS 458.93) as the outgroup. Strains isolated in this study are indicated in red.

In the phylogenetic tree of the FtorSC containing 14 *Fusarium* isolates, the strain *Fusarium venenatum* (CBS 458.93) served as the outgroup. The alignment comprised 5,084 concatenated nucleotide bases, viz., 1–547 (ITS), 548–1324 (LSU), 1325–2906 (*rpb1*), 2907–4668 (*rpb2*), and 4669–5084 (*tef1*). Among these sequences, 4,165 characters were constant, 431 characters were variable and parsimony-uninformative, and 488 characters were parsimony-informative. The topologies obtained from the ML and BI analyses were consistent; therefore, the ML tree is shown in this study. The 13 tested strains were identified as five species based on the phylogenetic analysis of the ITS, LSU, *rpb1*, *rpb2*, and *tef1* genes. In the BI analysis, the alignment contained 575 unique site patterns (ITS: 69; LSU: 26; *rpb1*: 159; *rpb2*: 193; *tef1*: 128). BI analysis was conducted for 20,000 generations, yielding 3,002 consensus trees (Fig. 2; BIPP ≥ 0.95 is displayed as the first value). In the phylogenetic tree, *Fusarium castanopsidicola* (SAUCC 14250-1) formed a well-supported clade (MLBS/BIPP = 100%/1.0) sister to *F. verae* (MST F23626*) within the FtorSC.

### Taxonomy

#### Fusarium castanopsidicola

Taxon classificationFungiHypocrealesNectriaceae

J.W. Zhang & X.G. Zhang sp. nov.

AB30FBFA-7FF4-5E43-81AE-6361B5355E66

864476

Fig. 3

##### Etymology.

Referring to the host genus, *Castanopsis*, from which the holotype was isolated.

**Figure 3.**
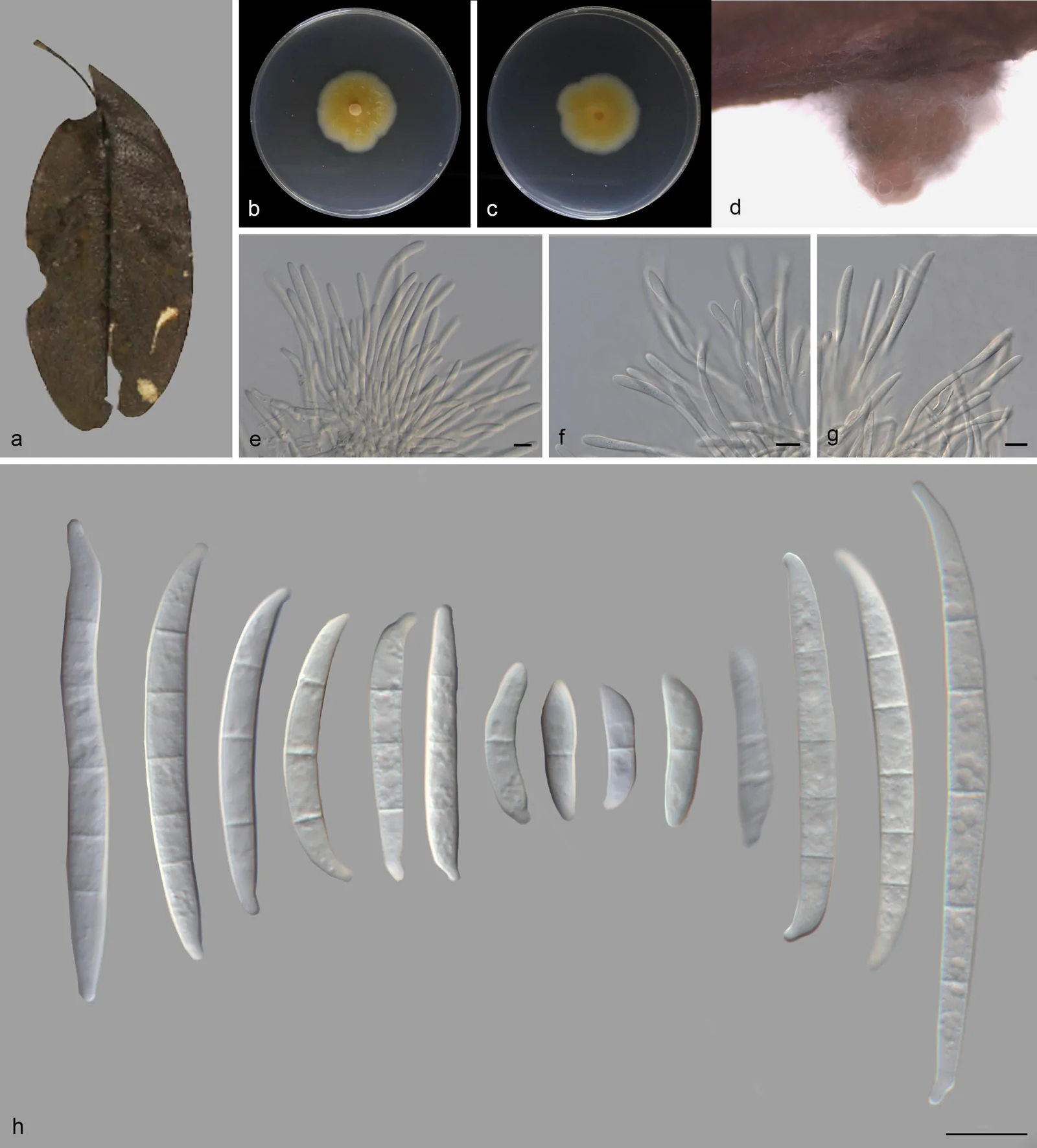
*Fusarium castanopsidicola* (ex-type CGMCC3.40253) **a**. Leaf of host plant; **b, c**. Upper and reverse views of cultures on PDA after 7 days of incubation at 25 °C; **d**. Sporodochia on carnation leaves; **e–g**. Sporodochial conidiophores; **h**. sporodochial conidia. Scale bars: 10 μm (**e–h**).

##### Typus.

China • Hainan Province, Wuzhishan City, Wuzhishan National Nature Reserve, on leaves of *Castanopsis* sp. 02 December 2024, Y.L. Wang (HSAUP 14250-1, holotype), ex-holotype culture CGMCC3.40253 = SAUCC 14250-1.

##### Description.

Aerial conidiophores and conidia were not observed. Sporodochia cinnamon, formed abundantly on surface of carnation leaves. Sporodochial conidiophores densely and irregularly branched, 28.3–51.3 × 3.1–5.4 μm (av. = 41.5 ± 6.7 × 4.0 ± 0.7 μm, *n* = 25), bearing a single phialide apically; sporodochial phialides monophialidic, subulate to subcylindrical, 17.7–34.3 × 2.2–4.2 μm (av. = 25.3 ± 5.8 × 3.4 ± 0.6 μm, *n* = 22), smooth, thin-walled, with a short-flared apical collarette; sporodochial conidia falcate, curved dorsiventrally, straight to slightly curved, tapering towards both ends, apical cell blunt to curved, basal cell papillate and poorly to well-developed, foot-shaped, 1–5-septate, hyaline, smooth, and thin-walled; 1-septate conidia: 15.9–20.1 × 3.8–5.2 μm (av. = 19.1 ± 1.8 × 4.3 ± 0.5 μm, *n* = 6); 2-septate conidia: 24.0–35.7 × 3.5–5.1 μm (av. 28.6 ± 4.8 × 4.3 ± 0.6 μm, *n* = 5); 3-septate conidia: 28.7–52.2 × 3.7–6.0 μm (av. 38.9 ± 7.2 × 5.0 ± 0.6 μm, *n* = 26); 4-septate conidia: 46.2–59.2 × 3.6–5.6 μm (av. 51.0 ± 5.4 × 4.9 ± 0.7 μm, *n* = 16); 5-septate conidia: 49.2–78.3 × 3.9–6.0 μm (av. 61.7 ± 9.4 × 4.9 ± 0.8 μm, *n* = 22). Chlamydospores not observed.

##### GenBank.

*CaM*: PZ621802; *tub2*: PZ706462.

##### Culture characteristics.

Colonies on PDA incubated at 25 °C in the dark, reaching 34–37 mm in diameter in 7 d; surface and reverse with a luteous central area, margin white, flat, odor absent.

##### Notes.

*Fusarium castanopsidicola* is introduced as a new species of the FtorSC based on a strain obtained from *Castanopsis* sp. in China. This species is phylogenetically closely related to *F. verae*. *Fusarium castanopsidicola* shows clear differences from *F. verae* in *tef1* sequences (6/286 bp differences) and *rpb2* sequences (24/1744 bp differences). Morphological characteristics of *F. verae* are not available (Tan et al. 2025).

#### Fusarium malloti

Taxon classificationFungiHypocrealesNectriaceae

J.W. Zhang & X.G. Zhang sp. nov.

FC5C9898-B74A-50B9-98A3-F7251B0B97F5

864475

Fig. 4

##### Etymology.

Referring to the host genus, *Mallotus*, from which the holotype was isolated.

**Figure 4.**
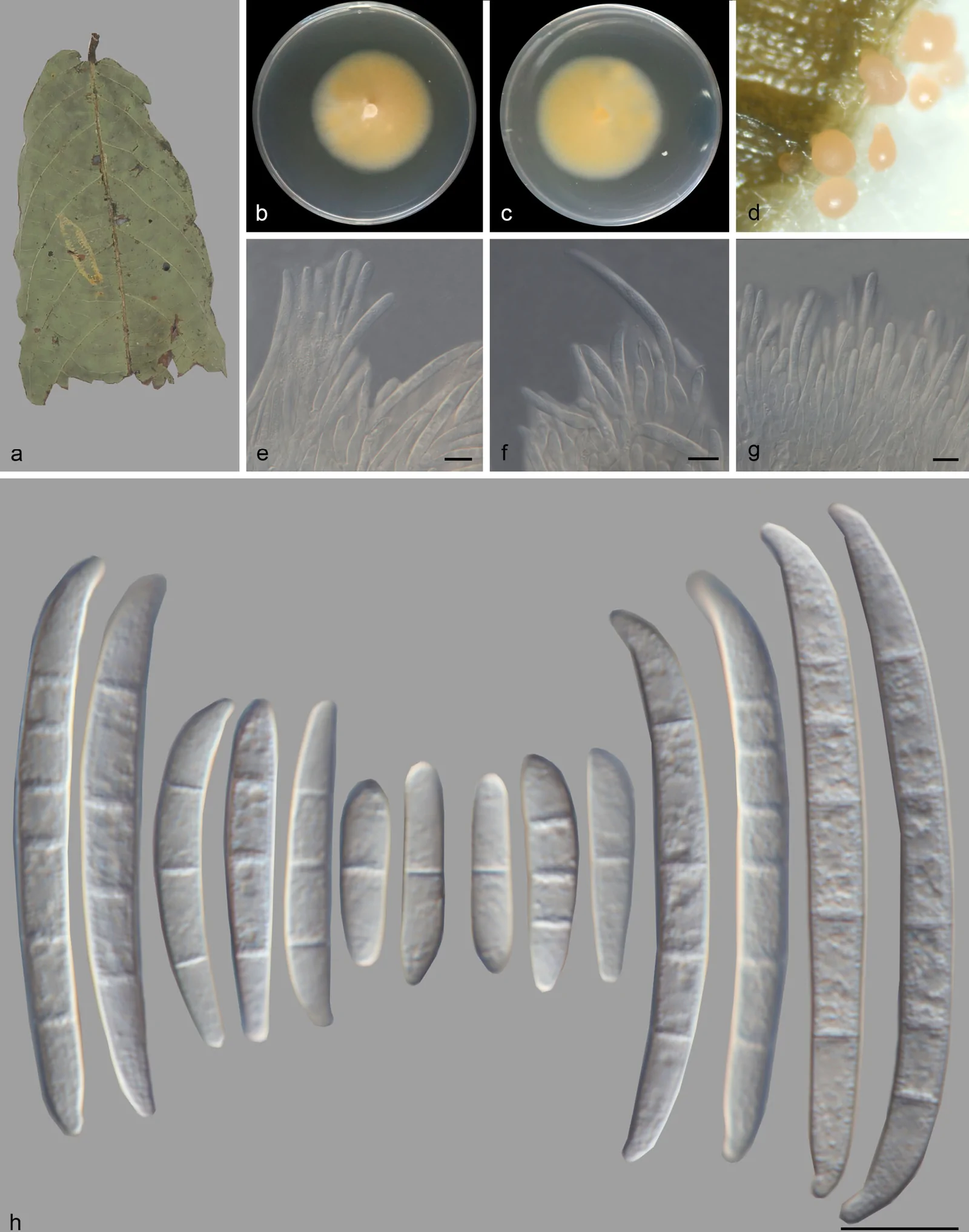
*Fusarium malloti* (ex-type CGMCC3.40250) **a**. Leaf of host plant; **b, c**. Upper and reverse views of cultures on PDA after 7 days of incubation at 25 °C; **d**. Sporodochia on carnation leaves; **e–g**. Sporodochial conidiophores; **h**. Sporodochial conidia. Scale bars: 10 μm (**e–h**).

##### Typus.

China • Hainan Province, Changjiang Li Autonomous County, Qicha Town, on leaves of *Mallotus japonicus*, 02 April 2023, Y.L. Wang (HSAUP 3317C-1C, holotype), ex-holotype culture CGMCC3.40250 = SAUCC 3317C-1C.

##### Description.

Aerial conidiophores and conidia were not observed. Sporodochia salmon, formed abundantly on surface of carnation leaves. Sporodochial conidiophores densely and irregularly branched, 11.0–17.8 × 3.7–5.1 μm (av. = 15.0 ± 2.3 × 4.4 ± 0.4 μm, *n* = 19), apically bearing 1–2 phialides; sporodochial phialides monophialidic, subulate to subcylindrical, 4.5–17.6 × 2.2–3.9 μm (av. = 9.6 ± 4.1 × 3.2 ± 0.6 μm, *n* = 18), smooth, thin-walled, with a short-flared apical collarette; sporodochial conidia falcate, curved dorsiventrally, straight to slightly curved, tapering towards both ends, apical cell blunt to curved, basal cell obtuse and poorly to well-developed, foot-shaped, 1–6-septate (2-septate absent), hyaline, smooth, and thin-walled; 1-septate conidia: 16.4–20.9 × 2.9–4.0 μm (av. = 18.8 ± 1.9 × 3.4 ± 0.5 μm, *n* = 4); 3-septate conidia: 21.9–54.9 × 3.7–4.8 μm (av. 36.0 ± 10.2 × 4.1 ± 0.3 μm, *n* = 18); 4-septate conidia: 39.5–58.7 × 3.4–6.5 μm (av. 48.9 ± 7.3 × 4.8 ± 0.9 μm, *n* = 20); 5-septate conidia: 52.5–65.0 × 3.4–6.0 μm (av. 57.1 ± 3.4 × 4.8 ± 0.9 μm, *n* = 23); 6-septate conidia: 47.7–51.2 × 3.9–5.3 μm (av. 49.4 ± 2.5 × 4.6 ± 1.0 μm, *n* = 2). Chlamydospores not observed.

##### Culture characteristics.

Colonies on PDA incubated at 25 °C in the dark, reaching 49–51 mm in diameter in 7 d; surface and reverse pale luteous, flat, odor absent.

##### Notes.

*Fusarium malloti* is introduced as a new species of the FLSC based on a strain obtained from *Mallotus japonicus* in China. This species is phylogenetically closely related to *F. lateritium*. *Fusarium malloti* can be distinguished from *F. lateritium* by the septation of sporodochial conidia (1–6-septate [2-septate absent] vs. 3–6[–9]-septate) and the size of 6-septate sporodochial conidia (47.7–51.2 × 3.9–5.3 μm vs. [70.0–]75.3–78.9[–82.0] × 5.0–6.5 μm) (Costa et al. 2024). Furthermore, *F. malloti* shows clear differences from *F. lateritium* in *tef1* sequences (8 bp differences) and *rpb2* sequences (14 bp differences, parts 1 and 2). It also differs in *tub2* sequences (2 bp differences).

## Discussion

In the present study, species delimitation within the FLSC was primarily based on multilocus phylogenetic analyses using *tef1*, *tub2*, *rpb2*, and *CaM* sequences. These markers were selected following previous studies that demonstrated their high discriminatory power in *Fusarium* taxonomy (Suwannarach et al. 2023; Costa et al. 2024). Consistent with these reports, the data showed that *tef1* and *rpb2* provided the best resolution for distinguishing closely related species within the FLSC (O’Donnell 2000; O’Donnell et al. 2013; Crous et al. 2021; He et al. 2024). Currently, more than 1,907 epithets of *Fusarium* have been listed in Index Fungorum (https://www.indexfungorum.org). The FLSC, which initially comprised only three species (*F. lateritium*, *F. stilboides*, and *F. sarcochroum*) when it was first established (O’Donnell et al. 2013), has since expanded to 29 phylogenetic species (Costa et al. 2024). This substantial increase highlights that the species richness of the FLSC remains underestimated, particularly in understudied regions and hosts. Within the FLSC, a novel species, *F. malloti*, was recovered from *Mallotus japonicus* and resolved as a distinct lineage. However, given that only a limited number of strains were obtained, further sampling is needed to clarify whether this fungus plays a substantial ecological role in Asian forest ecosystems.

Information on secondary metabolite production by FLSC species remains scarce. Although genome sequences are available for representative strains of *F. sarcochroum* and preliminary investigations have revealed gene clusters potentially linked to sphinganine-analog metabolites (SAMs) (Kim et al. 2020), the metabolic potential of most species in this complex has not been examined. The ecological flexibility of FLSC members is further underscored by a recent finding that an F-box protein (Flfbp1) in endophytic *F. lateritium* acts as a molecular switch between symbiosis and pathogenesis via β-carboline-mediated disruption of auxin homeostasis (Li et al. 2026). Future genomic and metabolomic analyses of *F. malloti* could reveal whether it harbors similar biosynthetic gene clusters, thereby providing insights into its ecological functions and potential biotechnological applications.

The FtorSC was established based on comparative studies of canker pathogens from *Torreya taxifolia* in Florida and *Zanthoxylum bungeanum* in China, initially comprising three core species: *F. torreyae*, *F. zanthoxyli*, and *F. continuum* (Zhou et al. 2016). With the subsequent discovery of *F. verae* from soil in New South Wales, Australia (Tan et al. 2025), the complex now comprises four species. In the present study, phylogenetic analyses based on ITS, LSU, *rpb1*, *rpb2*, and *tef1* sequences resolved a novel species, *F. castanopsidicola*, obtained from *Castanopsis* sp., as a distinct lineage within the FtorSC. The isolation of this strain from *Castanopsis (Fagaceae)* adds a new woody host to the known host range of the FtorSC. However, given that only a limited number of strains were obtained, further sampling is needed to determine the true ecological role of this species on *Castanopsis*. These findings also raise questions about the metabolic potential of FtorSC members, which remains largely unexplored.

## Supplementary Material

XML Treatment for Fusarium castanopsidicola

XML Treatment for Fusarium malloti

## Additional information

### Artificial Intelligence (AI) use

The authors accept full responsibility for the content of the manuscript, including the disclosure of any use of AI.

No AI tools were used in the preparation of this manuscript.

### Author ORCIDs

J. Zhang https://orcid.org/0009-0006-5134-1747

Y. Wang https://orcid.org/0009-0009-4029-2734

X. Zhang https://orcid.org/0000-0001-9733-8494

### Data availability

All of the data that support the findings of this study are available in the main text or Supplementary Information.

## Supplementary materials

Supplementary material 1 Supplementary information 1 Data type docx Explanation note Single-locus phylograms of the FLSC based on alignments of the *tef1*, *tub2*, and *rpb2* regions, respectively. Each tree is rooted with *Fusarium sublunatum* as the outgroup. MLBS ≥ 70% are shown above the nodes. Strains isolated in this study are indicated in red. https://binary.pensoft.net/file/1793864This dataset is made available under the Open Database License (http://opendatacommons.org/licenses/odbl/1.0/). The Open Database License (ODbL) is a license agreement intended to allow users to freely share, modify, and use this Dataset while maintaining this same freedom for others, provided that the original source and author(s) are credited. Jiawang Zhang, Yaling Wang, Jinyang Yuan, Xiuguo Zhang

Supplementary material 2 Supplementary information 2 Data type docx Explanation note Single-locus phylograms of the FtorSC based on alignments of the ITS, LSU, *rpb1*, *rpb2*, and *tef1* regions, respectively. Each tree is rooted with *Fusarium venenatum* as the outgroup. MLBS ≥ 70% are shown above the nodes. Strains isolated in this study are indicated in red. https://binary.pensoft.net/file/1793865This dataset is made available under the Open Database License (http://opendatacommons.org/licenses/odbl/1.0/). The Open Database License (ODbL) is a license agreement intended to allow users to freely share, modify, and use this Dataset while maintaining this same freedom for others, provided that the original source and author(s) are credited. Jiawang Zhang, Yaling Wang, Jinyang Yuan, Xiuguo Zhang

Supplementary material 3 Supplementary information 3 Data type docx Explanation note Phylogenetic tree inferred from a maximum likelihood (RAxML) analysis based on concatenated *tef1*, *tub2*, *rpb2*, and *CaM* sequences of species from the FLSC. Those marked with an asterisk (*) in the tree are represented by ex-type or ex-epitype strains. RAxML bootstrap support values (MLBS ≥ 70%) and Bayesian inference posterior probabilities (BIPP ≥ 0.90) are shown at the nodes. The tree was rooted with *Fusarium sublunatum* (CBS 189.34) as the outgroup. Strains isolated in this study are indicated in red. https://binary.pensoft.net/file/1793866This dataset is made available under the Open Database License (http://opendatacommons.org/licenses/odbl/1.0/). The Open Database License (ODbL) is a license agreement intended to allow users to freely share, modify, and use this Dataset while maintaining this same freedom for others, provided that the original source and author(s) are credited. Jiawang Zhang, Yaling Wang, Jinyang Yuan, Xiuguo Zhang
