# Supplementary material for: Morphological and phylogenetic analyses reveal new species of the Fusarium lateritium species complex and Fusarium torreyae species complex (Nectriaceae, Hypocreales) from Hainan Province, China
Source: MycoKeys. 2026 Sep 29;141:45–62. doi: 10.3897/mycokeys.141.209463 (PMC13643353; doi:10.3897/mycokeys.141.209463)
Supplement: Supplementary file 1 [file mycokeys-141-045-s001.docx]

**Supplementary Information 1:** Single-locus phylograms of the genus *Fusarium* based on alignments of the *tef1-α*, *tub2*, and *rpb2* regions, respectively. Each tree is rooted with *Fusarium venenatum* the outgroup. Strains isolated in this study were indicated in red.
