# Supplementary material for: Morphological and phylogenetic analyses reveal new species of the Fusarium lateritium species complex and Fusarium torreyae species complex (Nectriaceae, Hypocreales) from Hainan Province, China
Source: MycoKeys. 2026 Sep 29;141:45–62. doi: 10.3897/mycokeys.141.209463 (PMC13643353; doi:10.3897/mycokeys.141.209463)
Supplement: Supplementary file 3 [file mycokeys-141-045-s003.docx]

**Supplementary Information 3:** Phylogenetic tree inferred from a Maximum Likelihood (RAxML) analysis based on concatenated *tef1*, *tub2*, *rpb2*, and *CaM* sequences of species from the FLSC. Those marked “*” in the tree are represented as ex-type or ex-epitype strains. The RAxML Bootstrap support values (MLBS ≥ 70%) and Bayesian inference posterior probabilities (BIPP ≥ 0.90) were shown at the nodes. The tree was rooted to *Fusarium sublunatum* (CBS 189.34) as an outgroup. Strains isolated in this study were indicated in red.
